# Sixth Annual Session of the Southern Dental Association

**Published:** 1874-09

**Authors:** 


AMERICAN JOURNAL

OF

### DENTAL SCIENCE.

Vol. VIII. THIRD SERIES--SEPTEMBER, 1874. No. 5.

### ARTICLE I.

### *Sixth Annual Session of the Southern Dental Association.*

The sixth annual session of the Southern Dental Association assembled in the hall of the Polytechnic Institute, St. Louis, at 10 o'clock A. M., Tuesday, July 28th.

Dr. Robert Arthur, of Baltimore, president of the Association, called the meeting to order, and the exercises were opened with prayer by Rev. Dr. Linn, of St. Louis.

On the conclusion of the prayer, Dr. Isaiah Forbes delivered the following address of welcome:

*Gentlemen of the Southern Dental Association:*—In behalf of the dentists of St. Louis, I am called—not because of especial qualification, but as their senior in years—to the pleasant duty of welcoming you to this city. But not only in their behalf do I extend to you a cordial welcome; here are students in other branches of science who greet you as brothers. Here in this hall, the Academy of Natural Sciences, the Medical Society, the Historical Society, the Microscopical Society, and other kindred bodies, hold their meetings; and their respective libraries and museums, well worthy of a special visit; with the library of the Public School Association in this building, and by Mr. Bailey, the librarian, placed at your disposal during your stay. The

Mercantile Library Association, through its librarian, Mr. Dyer, has extended to you the free use of its extensive and valuable collections.

The press, zealous champion of truth and fearless antagonist of error, offers its columns for the publication of your proceedings, and heartily welcomes you to the city. And the powerful body of merchants, known by their enterprise to the furthest limits of this country, give you a cordial greeting. Manufactures, at once the pupil and servant of science, will invite you to observe in foundery or factory the wonderful progress of a city destined soon to equal, if not surpass, in its industry any other on this continent. In a word, St. Louis welcomes you with pleasure.

Thirty years ago a mere river town of 20,000 souls, and now a growing city of half a million inhabitants. It will be the aim of your professional brothers in this city, whereever your duties permit, to show you its beauties and objects of interest, and to make your visit one which I trust you will not be unwilling to repeat.

Permit me to add that the charms of scientific inquiry at these meetings are enhanced to me, at least, by the delight of personal intercourse. At the national convention of dentists, which met at Hope Chapel, New York, I met for the first time, many from whose unfailing fountains of thought I had for years gladly refreshed myself.

As star answers to star acro-s the wide expanse of the heavens, so each student in any branch of science, though widely separated from others of kindred pursuits, sends from his closet the rays of thought, the fruits of discovery, the records of experience, which other students far away hail with gladness, and like the stars the true followers of science send forth their rays of truth generously and ungrudgingly, glad if they reach not only worthy students of honest inquiry, but some darkened and rayless humbugs as well, who are thus enabled to shine a little with borrowed light.

In ours, as in other branches of science, the true student seeks the well-being and happiness of mankind. Not for any promised gain does he seek to hide the discoveries he makes, but, on the contrary, by associations such as this he gladly imparts them as freely as possible to others who may use them for the happiness of men. And in these gatherings we gain, not only by the interchange of thought, but by personal influence and example of our noblest associates. How great and lasting is that influence we rarely realize. The man of knightly fidelity and lofty patience never fails to leave his impress.

Often when operating we meet vexatious difficulties, which to me, as doubtless to others, are sometimes trying to the temper; defective instruments or foil, a nervous patient, weariness in the patient or myself have sometimes strained my self-control to the utmost. But if I turn my head, and see upon my walls the portrait of Dr. Arthur, its benignant expression soothes like magic; the recollection of your lofty patience, sir, calls back my wavering mastery of weary nerves, and I resume my task with comfort and success.

Gladly, gentlemen, in behalf of my professional brethren, and in behalf of this solid city, I have the honor to welcome you, and trust your visit may prove both profitable and agreeable.

This address was cordially responded to by President Arthur in behalf of the Association, expressive of its high appreciation of the kindness extended to it, and the hope that the workings of the convention might redound to the general welfare of the profession.

The roll was then called, and the following members answered to their names:

Dr. Robert Arthur, Baltimore, President; Dr. James Johnson, second Vice-president, Staunton, Va.; Dr. Jas. F. Thompson, Recording Secretary, Fredericksburg, Va.; Dr. S. J. Cobb, Nashville, Tenn.; Dr. A. P. Gore, Baltimore, Dr. S. H. Henkel, Staunton, Va.; Dr. Jas. S. Knapp, New Orleans; Dr. W. H. Morgan, Nashville, Tenn.; Dr. W. N. Morrison, St. Louis; Dr. W. G. Redman, Louisville, Ky.; Dr. J. R. Walker, New Orleans; Dr. W. T. Arrington, Memphis, Tennessee.

On motion, the reading of the minutes of the last meeting was dispensed with, they having been published in the proceedings of the Association.

Dr. Robert Arthur then read a lengthy essay on the present condition of dental science and the best means of advancing its true interests. He deprecated the idea that seemed to prevail in medical colleges and among medical gentlemen generally, that dentistry was merely a mechanical occupation, and that the dental profession knew no more about diseases of the teeth than they did. He considered dental surgery as one of the highest specialties in the great art of medicine. True, a great portion of the work of a dentist depended upon mechanical dexterity. No knowledge of physiology, chemistry, or therapeutics is of the slightest use to render a man fitted to perform the operation of filling teeth. Let an individual have natural or acquired manual dexterity, and he has appliances and material of such superior quality on hand that he can readily be taught to perform many of those operations in an entirely efficient and satisfactory manner. For many of the simplest operations of filling teeth, but little mechanical ability is required, and even for the very best operations a certain amount of manual dexterity, patience, perseverance, and careful attention to detail will go far towards accomplishing good results.

*The extraction of teeth does not require, as is generally supposed, any medical knowledge, nor even an acquaintance with the anatomy of the parts concerned. There is very little risk of breaking the jaw, as was formerly supposed to be likely to happen in the hands of an unskilful operator.*

But the better class of those engaged in dental pursuits, take a broader and more elevated view of their calling than these mechanical operations, and consider it worthy of a higher rank than simply that of an artistic calling.

First. It is concerned with the care, locally, of important parts of the organism, more subject to diseased conditions than any other, many of which are extremely painful, and some of the gravest character.

Second. The effect of diseases of the teeth are not confined to themselves nor to important parts adjacent thereto, but in many cases seriously affect the whole system, not unfrequently leading to great suffering, to general disorder, and to the abridgment of life. On the other hand certain conditions of the general system seriously impair the healthy condition of the teeth.

Third. So far as general disorder is occasioned by the teeth, the diseased condition of the latter deserve special attention and study.

The speaker deduced from these propositions that medical men are ignorant of the pathological conditions of the teeth, except in a superficial degree; that they give too little attention to the influence of the teeth upon the general system, and that it is important to the interest of the community that there should be a class of medical specialists capable of estimating fully the influence of diseased teeth upon the general system, and *vice versa.*

Drs. S. H. Henkel, James S. Knapp and W. H. Morgan, were appointed a committee on membership, to report at the afternoon session.

Dr. W. G. Redman, of Louisville, was appointed secretary pro tem.

Dr. J. S. King and Dr. R. R. Freeman, of Nashville, and Dr. Welchens, of Pennsylvania, presented their credentials, and were admitted to seats.

Dr. James Johnson, of Staunton, Va., offered the following which was adopted:

*Resolved*, That a cordial invitation be extended to the dental and medical professions of the city and surrounding country to occupy seats on the floor during the meetings of this Association.

Dr. Walker, of New Orleans, offered a resolution fixing the hours of meeting, but the question was laid over until the afternoon session, and the convention adjourned until 2 o'clock.

AFTERNOON SESSION.

At about half-past 2 o'clock the afternoon session opened, the same members as in the morning being present.

The first order of business being the settlement of the question as to hours of meeting, the executive committee rceommended the following: For the first two days, clinics and examination of dental appliances. Morning session from 10 to 12, afternoon session from 2 to 6, evening session from 8 to 10. For the remaining days, clinics from 8 to 10, morning session from 10 A. M. to 2 P. M., evening session from 8 to 10. These hours were considered good, and were adopted as the choice of the Association.

A communication from Messrs. Geo. Knapp & Co., inviting the Association to visit the *Republican* office and witness the operation of the Walter press, was read, and the invitation accepted.

An invitation from Mayor Brown, for the Association to take an excursion upon the harbor boat, on Thursday afternoon, for the purpose of visiting the Vulcan Iron Works, Water Works, and other features of interest, was also read and accepted.

The committee on mechanical dentistry also made its report in the form of an exhaustive paper on the subject of the manufacture of teeth. It set forth that sets of teeth manufactured of cheap material were always the most costly in the end, and that any dentist who recommends to his patients an article of this kind, is not doing his duty as a professional man. Collodion was recommended as being by far the best and most satisfactory base that can be used. The paper went on to insist that more time and labor be given to experiments and investigations in the laboratory, as at present the science is greatly dependent for its progress on the works of men who have no connection whatever with dentistry, and who cannot, of course, take that interest in the branch that a professional would.

The report was accepted and filed among the records of the Association.

Dr. Walker, of New Orleans, stated that he had always been an uncompromising opponent of the use of rubber as a base for artificial teeth, and another year's experience had only confirmed him in his opinion on the subject. He preferred collodion above all other materials for that purpose.

Dr. Welchens, of Pennsylvania, stated that he and his friends had attended this Convention for the purpose of learning the opinions of the Western dentists on the use of rubber as a base. The dentists in this part of the country have found it impossible to entirely give up the use of rubber, but its use has certainly driven science out of the laboratory, by admitting to the practice of mechanical dentistry unskilled and inexperienced persons, it requiring no long experience or skill to enable anyone to make a comparatively good job on a rubber base. This state of affairs results in a great slaughter of the teeth, and has reduced the prices of dentistry to an unpleasantly low figure. For these reasons the use of rubber is undesirable, but at the same time it is absolutely impossible in some portions of Pennsylvania to do without it. People having been educated to the use of cheap rubber sets, it is a very hard matter to convince them that gold and silver bases are so much more desirable as to warrant the great increase in price. Hence, an operator in Pennsylvania must be prepared to work on all the different materials, else he will loose a large portion of his patronage.

The committee on membership reported favorably upon the following named applicants for admission to the Association, and they were all elected by a unanimous vote: H. Judd, M. D., D. D. S., St. Louis, Mo.; H. J. McKellops, D. D. S., St. Louis, Mo.; R. J. Porre, D. D. S., St. Louis, Mo.; J. B. Newly, D. D. S., St. Louis, Mo.; F. Forbes, D. D. S., St. Louis, Mo.; C. Stoddard Smith, D. D. S., Springfield Ill.; R. E. McReynolds, D. D. S., Macon, Ga.; M. A. Bartleson, D. D. S., St. Louis, Mo.; W. H. Eames, D. D. S., St. Louis, Mo; Jas. Knapp, D. D. S., New Orleans.

After five minutes recess to allow the newly elected members to sign the constitution, the discussion of mechanical dentistry was resumed.

Dr. Morgan, of Nashville, stated that his observations as to the effect of a rubber base upon the mouth did not agree with those of Dr. Walker. He did not believe that it injured the mouth any more than silver, and even gold had a very slight advantage over it. In support of this he exhibited his own mouth, which, although it had worn rubber for nearly two years, he declared to be perfectly sound.

Dr. Walker replied that he had given the matter much and close study, and was satisfied that his position was correct. His own wife had worn rubber for three years, and the effect was to ruin her health. He was not convinced that this was the cause until he tried an experiment upon his own mouth with a like effect. By watching the effects of the same agency in numerous cases, he placed the matter beyond all doubt. Almost universally he noticed that it not only made the mouth sore, but it had such an effect upon health, as to reduce the weight of the person very considerably, the person returning to his normal condition as soon as the cause was removed. He gave some of the symptoms of the disease produced, but stated that he had never analyzed the rubber after vulcanizing to ascertain the deleterious effects.

Dr. Judd stated that the question was one of great importance to the entire world. He had given the effects of rubber a great deal of attention ever since the first cry was raised against it. He had found that the red rubber plate is far more injurious than black rubber, and cited an instance in which the two rubbers were mixed, the part touched by the black being comparatively healthy, while that touched by the red rubber was greatly irritated, and even inflamed. He knew of several cases where the disease had extended to even the bones of the mouth, completely destroying them. While he believed that in different climates the degree of injury may vary, he was satisfied that red rubber always produces more or less irritation, inflammatory action, or disease. He had seen a number of cases where the mouth was greatly diseased by rubber plates, and when metal plates were substituted the parts recovered their healthy condition. Why should not red rubber have this effect? Let any one, no matter how healthy, place a small quantity of red sulphuret of mercury in his mouth and allow it to remain for a while, and he will find the parts to become almost immediately affected. The red sulphuret of mercury cannot be covered up by the ingredients of red rubber so as not to have this deleterious effect. With black rubber he had not had such an extensive experience, but he was satisfied that it had not such severe effect as red rubber.

Dr. Johnson said his experience agreed exactly with that of Dr. Judd. He had seen some very serious cases of disease which had arisen from red rubber, and he has seen one or two cases of several years' standing, which were cured by simply substituting a metal for a rubber plate. He had years ago given up the use of rubber entirely, and would never return to it.

Dr. Knapp agreed that rubber should not be used for permanent plates, but held that sometimes it is necessary for irregular teeth, and for temporary plates. He had also found that red rubber was far more deleterious than black rubber. He could not understand, however, how the mercury could be released from the saliva to such an extent that it brings on disease.

Dr. Morgan stated that he believed that the majority of diseases of this nature arise, not from the material of the plates, but from carelessness or uncleanliness on the part of the wearer. He had known of a number of cases of very serious disease in mouths where gold plates were used, and in one of these he had substituted a rubber plate with most gratifying effects. In the cases mentioned by the gentlemen who recently spoke, it is not impossible that if the plates had been silver or gold instead of rubber, the disease would have been much worse.

Dr. Cobb, of Nashville, believed that disease arises more from the fact of a foreign substance being in contact with the mucous membrane than from the nature of that foreign substance. He admitted, however, that there are more cases of disease with rubber plates than from silver or gold. He deplored the apparent lack of disposition to investigate this matter to the fullest extent. He would be glad that some member had so far studied the matter as to be able to give the component parts of vulcanized rubber, which being done the cause of disease could be more readily arrived at. He did not think it advisable to reject rubber altogether, however, until some scientific analysis has absolutely proven it to be the promoter of disease. So long as there are poor people, we will need a cheap substance for the manufacture of bases for teeth, and if rubber is proven to have nothing in it of a deleterious nature, then it is truly a blessing to the profession.

Dr. Arthur agreed that the cases of disease where red rubber is used, are more numerous than where any other material is worn, but he also regretted that with all the appliances so convenient, this matter has never been scientifically investigated. He, therefore, moved that a committee be appointed with instructions to subject vulcanized rubber to a chemical analysis, and to report at the next meeting. The motion carried, and Drs. Judd, Eames and McKellops, of St. Louis, and Dr. Summers, of Nashville, were appointed as members of that committee.

Dr. Knapp said that another cause for disease might be found in the fact that, where rubber is used, the mucous membrane is more perfectly or entirely covered than where metal is used, hence the inflammation is greater.

Dr. Forbes said that he considered that very often the effect of the plate depended upon the physical idiosyncracies of the person wearing it, and often the fact that some of the teeth have metal fillings, on which the mercury in the rubber acts, explains the disease. He deprecated the common custom of denominating low-priced articles as "cheap." With him, that which serves the best purpose, no matter what its price, is cheapest. He hoped that hereafter the "white" members of the profession would drop this improper use of the expression.

The subject was then dropped.

A letter from Prof. McLean, of New Orleans, announcing his regrets that he was unable to attend the meeting, was read and accepted.

The following gentlemen were appointed a committee for the performance of clinical operations at the office, No. 904 Chesnut Street, this morning from the hours of 8 to 10: Drs. Knapp, Arrington and Walker.

The chairman of the histology and microscopy committee was not present, but the written report was received. As it was of a very trying order of chirography, its reading was postponed until to-day, in order to give the secretary an opportunity to practice on it.

A motion to invite all members of the medical and dental profession of the city to attend the meetings of the Association was, after some sharp opposition, lost.

A motion to invite the physicians of the city to the same privileges was also fatally opposed.

Dr. Redmond offered an amendment to the constitution, providing that members shall be required to pay annual dues for only such years as they may attend the meeting of the Association. This provision is to apply from the date of organization. Dr. Redmond said that he had acted as treasurer for a long time, and he knew that the feeling of a majority of the members favored this proposition.

Some discussion arose in which it was argued that this would be beneficial, in the fact that sometimes members remain away from necessity several years, and then drop out entirely, because to come back would be to have to pay back fees without receiving any benefit therefrom. This change being adopted they would not drop out, and thus the membership would be kept up to a good standard. The amendment was adopted.

Dr. Redmond also moved that the name be changed to "The National Dental Association."

Dr. Morgan said that the necessity which gave rise to the old name has died away, and as the name justifies the common inference that it is a sectional institution, while it is desirable to do away with this idea. Hence he favored the change.

Dr. Johnson opposed the proposition. He held that it would not benefit the Association or increase its membership to make the change. Dr. Redmond said he had been unable to induce any dentists from his State to attend because of the impression that the Association was a Southern one.

The amendment was laid over for discussion at some future year.

Dr. Judd read an essay upon the subject of "Deciduous Teeth," showing that it is just as important to give good care to the deciduous as to the permanent teeth, and that the pain of losing them is, to the little folks, as great as that of losing permanent teeth, to adults. If the deciduous teeth are carelessly treated, and if no efforts are given to preserve them, the result is not only the temporary pain which the child suffers, but in the permanent malformation of the jaw, which contracts on the premature removal of some of the teeth, and results in irregularity of the permanent teeth, and in great injury to health and physical development. He then went on to state how deciduous teeth should be cared for while perfect, and the manner of treatment if diseased.

The paper was accepted and ordered published.

Drs. Walker, Knapp, Morgan, and Johnson, and Arthur, heartily indorsed the views of the paper, declaring that almost universally the importance of caring for children's teeth is unrecognized, and that the bad effects of this want of care remain with the child through life. The teeth should be kept scrupulously clean, the interstices should be kept open with the file and disk, and every effort should be used to preserve the deciduous teeth until they are removed by natural process. The result will always be good health in the child, perfect development of body, and a regular and durable set of permanent teeth.

EVENING SESSION.

The Association met at 8 o'clock, and resumed the discussion of the treatment of deciduous teeth.

Dr. Morgan said that he was opposed to the plan proposed by Dr. Johnson, to the effect that children's teeth should, if very close together, be separated by files or disks. He believed that such a practice would be barbarous. So long as a portion of the body is not diseased, it is not in need of surgical interference. Even if the teeth are close together at first, the natural enlargement of the jaw will separate them.

The committee on members presented the name of Edgar Parks as a candidate for admission to the Association, and he was elected to membership by a unanimous vote.

Dr. Cobb agreed with the views of Dr. Morgan, and thought the plan of separation to be too much of the order of anticipation. He dwelt at length on the necessity of great care in the treatment of children's teeth. He did believe, however, that if any evidences of decay are seen where teeth touch each other, the decayed portions should be cut out.

Dr. Knapp held that to improve the quality of the teeth, we should go back to primary causes, and advise mothers to eat such food during gestation as will contribute to the construction of sound teeth. Again, this same cause of using proper food should be urged upon young children, as the quality of teeth depends upon the kind of food eaten. He, like the other two gentlemen just seated, deprecated the separation of well-formed teeth in children, so long as they remain sound, no matter how close together they may be. He believed in preserving the first teeth as long as possible —by filling, if necessary, in order that the proper shape of the mouth should be retained. The filling employed, if any, should be gold, if it is to remain long, but if the teeth are to be shed soon, amalgam can with propriety be used.

Dr. Freeman, of Nashville, held that to separate teeth with instruments, would be to render the parents of the child careless, as they would think that the dentist has performed an operation which relieves them of all responsibility. He believed in filling a deciduous tooth just as soon as it began to show signs of decay.

Dr. Judd made a few remarks in regard to the treatment of children in the chair. He stated that it is his practice, at the child's first sitting, to only rub one or two smooth instruments over the teeth and gums—to dip some cotton in water and put it in the tooth, and to feign several operations without causing any pain, after which he lets the child go home. This removes from the child's mind the dread which a little one always has of going to a dentist, and he is perfectly willing to come back again and submit to an operation, painful though it be.

Dr. Cobb believed in this treatment, but held that no false representations should ever be made to a child, as he quickly discovers it, and it destroys his confidence for all time.

Dr. McKellops desired to see a clinic on this subject. He held that no amount of confidence will induce a little child to submit quietly to a painful operation. Sometimes the little one's teeth are so affected that they are even more painful than those of adults, and it is unreasonable to expect that they will submit to having those teeth treated with instruments without making resistance. He wanted to see this easy method of treating children practically demonstrated at a clinic.

Drs. Cobb, Walker, McKellops, Arthur and Judd, discussed the subject somewhat further, but want of space will not admit of a synopsis of their remarks here.

The committee on membership proposed Henry S. Chase, D. D. S., as a member, and he was elected by a unanimous vote.

The Association then took up the subject of "Irregularities in Permanent Teeth, their Cause and Treatment." Dr. Knapp said the cause as had been explained in the discussion just ended, existed in the premature removal of the first teeth, and in leaving the fangs of the first teeth too long in place. The manner of remedy was to apply a constant pressure upon the teeth which are out of line, thus bringing them gradually into place. It is a much more troublesome matter to bring to place teeth which protrude too far outward, than to bring those which protrude inside the arch to their proper line. He mentioned the dangers to be guarded against, and gave his method of operation in different cases of irregularities.

Dr. Walker said that each case of irregularity requires a special contrivance, and hence no universal rule could be laid down for their treatment. He gave the history of a very puzzling case which he once treated, where the upper teeth of the patient stuck straight out, and he had to rig up a scaffolding on the boy's face to get a proper purchase for righting the disagreeable wrong. It took three months to get the teeth to point in the proper direction.

Dr. Arthur also described the treatment by which a difficult case in his charge was being treated, a jack-screw playing a very prominent part in the correcting process, by being braced inside the arch against the tooth which had gone astray, and by a turn or two each day, enlarging the arch and forcing the tooth into position.

Dr. Morgan gave an interesting case of the same nature, which he also treated with a jack-screw. The case was a very obstinate one, and after exhausting the resources of mechanics, and building some very complex machines in the patient's mouth, he was compelled to give the case up in an imperfect condition.

Dr. Judd then took up the subject, and made a very exhaustive and interesting review of the different methods of overcoming irregularities, quoting authorities and comparing their recommendations with the result of his own observations. He did not believe in the custom in vogue in Europe of bringing the teeth into place by a sudden application of power. He did believe, however, that six days was ample time for correcting nearly any tooth.

Dr. Welchens told of the method adopted by a physician with whom he was acquainted, who had been very successful in twisting teeth into a proper position by a single wrench with the forceps. He had treated a number of cases this way without even causing noticeable inflammation.

The Association then adjourned until Wednesday morning.

---

SECOND DAY'S PROCEEDINGS.—MORNING SESSION.

The Convention came to order at 10 A. M., President Arthur in the chair.

Record of yesterday's proceedings read and approved.

*Irregularities of Teeth* being still open to discussion, Dr. Morrison exhibited some interesting models of cases of irregular teeth which came under his observation, one of a case of irregular deciduous molar, and the other of a superior canine. He explained to the Convention his manner of treatment.

Dr. Walker, of New Orleans, demonstrated on the blackboard a very simple method of bringing an angular incisor within the arch natural to it by the use of a simple brace and ring.

Dr. Forbes said in the case of irregularities, if the dentist had any test in the matter, it looked toward the personal appearance of the patient, especially if a young lady be in the chair. If the young lady have a sharp nose, square face, and retiring chin, no one would think of expanding the arches. He related two cases to the point. If a child has a large nose, and protruding chin, he would treat the irregularity in quite a different manner.

Dr. Chase, of St. Louis, said it required more than ordinary courage to tell a patient that it is necessary to loose three or four sound teeth. It would be better for nine tenths of humanity if at the age of fourteen they lost three or four of the bicuspids.

On motion, the subject of irregularities were passed.

*Operative Dentistry.*—The Association passed to the question of operative dentistry.

Dr. Arthur, President of the Association, was unanimously ceded the floor to discuss this subject. He was quite ready to enter into the subject. He had for many years been investigating the subject, and stood ready to defend his peculiar views.

Dr. Chase, of St. Louis, was fond of the truth, and liked to express it. He also liked to hear others express their convictions. He was in favor of extracting sound teeth instead of cutting away. In cutting away you deform more teeth than you do in extraction. If at the age of eleven or twelve years he found anything the matter with any of the bicuspids, he would ask the question, will this tooth be sound at the age of twenty-five? If not, extract it. He contended that blood circulates in the enamel. He was in favor of giving each tooth room by the extraction of the bicuspids or first molar. In the examination of a child's mouth it is necessary to look ahead and ask what will be the condition of the child's teeth at the age of twenty-five.

Dr. Arthur would not enter into any discussion with the last speaker concerning his peculiar views upon the enamel of the teeth. As soon as the permanent molars take their places, caries sets in. The period when these teeth should be extracted is very important. If taken out too soon the result may be disastrous. It had been said that he had advised the indiscriminate filling of teeth of children at any age. He never advised any such thing. How any one could read his work on the "Prevention of Decay of the Teeth" and say so he could not see.

Dr. Morgan was merely in search of the truth. He differed with the ideas of Dr. Arthur as expressed in his practice. His system contemplates the cutting away of the enamel and dentine without pain. Who is willing to exercise this doctrine? No one. Dr. Morgan selected several portions of Dr. Arthur's work, especially those parts relative to the preservation of the teeth, and pointed out what he contended were falacies. He denied that filling or plugging were the only methods of arresting decay, as asserted by Dr. Arthur.

Dr. Arthur would take all remarks made upon his book in the spirit of controversy and for the benefit of science. As regards the question of his having a hobby, what man would take up a subject which he knows to be all important, and is firmly convinced of its truth, but will advocate it with all his power? He would leave the full answer to the gentleman's argument until a future session.

Convention adjourned until 2 o'clock.

*Sight Seeing.*—During the recess at noon, the delegates availed themselves of the invitation of George Knapp & Co. to visit the *Republican* office and view the Walter press in operation. The Convention was shown through the different portions of the building, and the various departments of a newspaper office were explained to them.

AFTERNOON SESSION.

President Arthur called the Convention to order at 2:15.

*Electric Instruments.*—With the permission of the Convention, Dr. Arthur made some explanation of electric instruments, as used in dentistry. For this purpose the Dr. had fitted up an electric battery, supplied with instruments of various kinds for the filling and plugging of teeth. The same principle is used as in the case of the electric telegraph. The common horse shoe magnet is used. Applying the mallet of Dr. Jack to the battery, the instrument strikes some 3000 blows per minute. It is so constructed as to regulate the force of the blows, thus giving the operator perfect control over the machine.

The electric engine was next applied to the battery. The revolutions of this instrument amount to about 2,500 per minute. These two instruments are now considered the most perfect in use, and the president took great pleasure in recommending their use to the profession.

The name of Samuel Russell, D. D. S., of Lancaster, Pa., was proposed for membership, and, upon motion was unanimously elected.

*Operative Dentistry.*—Dr. S. J. Cobb, of Nashville, chairman of the committee on Operative Dentistry, made a report on the subject, which was read to the Convention. The report started out with laying down as an hypothesis that Americans as a nation were in many respects too fast; and that some dentists might be accused of the same, in many cases grave fault. It touches upon the relative merits of cohesive and non-cohesive gold as a filling for the teeth. The former was a modern article in the practice of dentistry; the latter is of old and tried reputation. The old method of using the non-cohesive is far more desirable than the cohesive. The report quoted various instances where the cohesive gold has not remained in the cavity of the tooth. Many other reasons were forwarded why the non-cohesive should be used. The report reviewed the Morrison engine and its uses, and the care with which operators should use such instruments. It also examined the use of oxychloride of zinc, creosote paste, as applied in the practice of dentistry.

A letter from Dr. Branch, a member of the Assciation, was read, regretting that circumstances forbade his being present.

*Dr. Arthur's Book.*—Having vacated the chair, Dr. Arthur came forward to answer to some of the strictures made by Dr. Morgan, of Nashville, upon his work. His work was indorsed by the leading practitioners of the promiinent cities in the country. A criticism in the *Missouri Medical Journal* had also indorsed it, although not agreeing in all that it had put forward. The subject treated in his work had cost him a life-time of study. Dr. Morgan denies the power of any man to foresee what the teeth of a child will be when it has attained manhood. He did not claim the gift of inspiration for himself, but he did claim as much common sense as the generality of men. It may be obtained by referring to the teeth of the parents, and their physical condition otherwise. If, on carefully examining the teeth of a child five years old, and the incisor teeth are found decayed, there are not five per cent. who will escape decayed teeth or caries of the teeth. If this practice advocated by him in his work is admitted as a sound one, it becomes the duty of the profession to adopt it immediately. As honorable men, they had to convince themselves and others that his system of practice was injurious, or they must adopt it. There was no way of getting out of the dilemma.

*Criticism.*—Dr. Judd, of St. Louis, would review a few points of the morning session. He would touch upon the indiscriminate extraction of the six year old molars. He would state that nineteen-twentieths of the dental profession did not hold any such doctrine, and especially the intelligent portion of the profession. As regards the work of Dr. Arthur, he understood that it was not claimed to be entirely new. This practice is as old as the practice of dentistry itself. It was allowed to fall into disuse. Dr. Arthur has revived this practice. During those years it had almost entirely fallen into disuse, and it was due to Dr. Arthur that this practice has again become so general. There was no doubt but a great many prominent practitioners use this system of practice much more frequently than they would have, had Dr. Arthur's work not appeared, although they are unwilling to go as far as the Doctor would carry them. Upon the rough surface of molars can be found fossi, or cavities, where decay does not exist, and which if left alone will go through life without decay. But it is a well known fact that some practitioners consider it a duty to fill every cavity which can be discovered upon the surface of the teeth. In this light, he did not agree with Dr. Arthur's work. It was time enough to fill the cavity when decay is found to exist. He would acknowledge that this work had modified his practice, and more frequently separated teeth than formerly.

Dr. Morgan regretted that his manner or his words would justify the president to characterize his opposition to his work as bitter. He always endeavored to avoid bitter feelings on all occasions. Dr. Arthur referred to his ability to tell whether the teeth of a child will decay or not. He would relate a case in which he could not. He might examine the teeth of a child twelve years old; some of the teeth may be decayed, and he defied him to determine whether the balance would decay or not. The child might die in a month, and, if he has separated those teeth, he has inflicted so much useless suffering upon humanity. This book was before the profession. If I am satisfied that it is incorrect, is it not my business to disprove it? If I go right along and practice my profession according to my own ideas, it is not with me to disprove it. On one page he says enamel has no power to resist decay, and on another that there is no question but well-formed enamel has the power to resist decay more than the dentine. The greatest fault he found with the book was that it recommended operations when there was no decay. He proposes to physic where there is no sickness.

Dr. Freeman would like to see the good portions of this book brought out just as well as its bad points. Doubtless as much acceptable matter could be found in it as has been found objectionable.

Dr. Knapp was of the opinion that there were a great many good things in the book, and it should be in the library of every practicing dentist.

Dr. Cobb thought we were all too apt to find fault with other people's work. The profession was under many obligations to Dr. Arthur for the book.

Dr. Forbes looked upon the book as one of the most fascinating in the annals of dentistry. He would have fallen a victim to this book had he not been taught the abuse of the file. Some time ago it was the fashion for ladies to have their front teeth standing apart. Many a time had he been called to the operating rooms to witness the sad effects of the abuse of the file.

On motion, the subject was postponed until next session.

Upon motion, the convention proceeded to select a place for holding the next annual meeting of the convention.

The first ballot resulted as follows: Nashville, 8; Memphis, 8; Atlanta, 4.

On the second ballot Memphis received 10 votes, and Nashville 11 votes. Whereupon Nashville was declared the place for holding next meeting.

*The Excursion.*—The convention concluded to proceed in a body, at 2½ o'clock, to the harbor boat, at the foot of Walnut Street, and accept the invitation of the Mayor to make an excursion on the river, and visit the Waterworks, Carondelet furnaces, etc.

Dr. Knapp moved that the time of holding next meeting be set for the second Tuesday in April.

Many of the members found it very inconvenient to attend the convention at that particular season of the year.

The motion was withdrawn. The time was set for the last Tuesday in July.

Dr. Freeman moved that the vote selecting Nashville for the next meeting be re-considered. Carried.

On motion, Memphis was unanimously chosen as the next place of meeting.

Convention adjourned until 8 o'clock.

EVENING SESSION.

The Secretary read a circular on the subject of the Barnum Memorial Fund, which was suggested by the

American Dental Association at its last meeting, at Put-in-Bay. The memorial was donated to Dr. Barnum for bestowing on the profession the well known "rubber dam."

On motion, the Association voted $100 to the fund.

The Committee on Publication reported the following accounts to have been made on behalf of the Association:

| | |
|---|---|
| Messrs J. Murphy & Co., balance for printing, | $ 2 25 |
| Dr. Samuel Welchens, - - - - - | 100 00 |
| Total, - - - - - - | $102 25 |

The report was received and referred to a special committee, consisting of Drs. Arrington, McKellops and Thompson.

*Operative Dentistry.*—Dr. Knapp was the first speaker. He performed all his operations after the old plan. He was an old fogy. He did not use the rubber dam, yet he did not condemn its use. He used gold in the form of cylinders, and he claimed that just as good work can be done with cylinders as with any other form. The doctor entered into quite a lengthy dissertation upon the manner of placing the cylinders in the cavity of the tooth about to be filled, and the mode of bringing lateral pressure so as to force the cylinders against the walls of the cavity. He claimed that for all ordinary filling this form of gold presented many advantages. He had used cylinders since 1851, so he should know something about them.

Dr. Arthur would say a few words about cohesive gold. This quality of gold has been before the profession for about eighteen years. That this adhesive quality was well known to good-workers for a long period, he had no doubt. This adhesive gold is nothing more than pure gold.

Dr. George S. Peck, of New York, manufacturer of dental gold, explained the qualities of gold. The greatest question was to get non-adhesive gold. The softest gold made is the Eureka gold made by himself. The purest gold is the most adhesive, and is consequently the safest.

Dr. Morgan said he could fill the cavities of teeth with adhesive gold in as short a time as with cylinders. He also took occasion to remark that in order to have gold cohesive it is not necessary to have it pure: He saw gold with six per cent. alloy and still cohesive.

Dr. McKellops did not agree with Dr. Knapp in his ideas on the subject of gold in the form of cyl nders. He was of opinion that he could take the adhesive gold, and with it reach every minute cavity of the tooth better than he could with cylinders. For fine work he preferred foil. The best foil for dental purposes was the roll foil Nos. 30 and 60.

Dr. Cobb, of Nashville, was in favor of the old form of cylinders. He believed that more teeth were preserved in the old way than the new. He also believed that more teeth can now be saved by using non-cohesive gold. Many who think they are making first-class operations are making failures.

Convention adjourned until 10 A. M. to-morrow.

---

THIRD DAY'S PROCEEDINGS.—MORNING SESSION.

The third day's meeting of the Dental Association was called to order at 10 o'clock A. M. After the minutes of the previous meeting had been read and approved, "Operative Dentistry" was announced as the question for discussion.

Dr. Knapp, of New Orleans, arose and said that he had witnessed that morning an operation in this branch of dentistry, in which machinery was used for grinding out the cavity of the tooth. The doctor stated that the performer consumed an hour at the work, and that he was satisfied it could have been done by hand in fifteen minutes.

Dr. Eames gave his experience with metals used in the practice of dentistry. He preferred gold to all other metals.

Dr. King of Nashville, considered himself discoverer of a new method of treating exposed nerves until very lately. His plan consists of a cap of zinc and creosote.

Several gentlemen spoke of the importance of the method, while others did not exactly condemn it, but looked upon it in rather a doubtful manner.

An amendment to the constitution allowing the Secretary fifty dollars for each sessions work, was adopted.

An amendment offered by Dr. Walker, altering article 7, section 5, of the constitution, was adopted.

Dr. Arthur gave blackboard illustrations of the latest manner of placing the pivot in teeth.

W. G. Kingsberry, of San Antonio, Texas, was admitted as a member of the Association. The Convention then adjourned to meet again at 8 P. M.

EVENING SESSION.

A paper on Histology and Microscopy by Dr. S. P. Cutler was read by the Secretary, the doctor speaking of ossification of the pulps, and other topics of the same nature. The paper was accepted. Dr. Walker arose and moved that one of the gentlemen present, who was familiar with Dr. Cutler's hand-writing, should read the paper. (Much merriment.) Dr. Arrington asked the gentleman from New Orleans if he'd been asleep, stating that the paper had just been read. The doctor begged to be excused, remarking that he had been to Carondelet during the day. Dr. Judd opened the discussion on the subject, speaking at length of the dental tubuli, of neuralgic affections, and of calification and dentine of the pulp. Dr. Knapp, of New Orleans, spoke of a case where he tried to remove the pulp, and of his investigation on dentine and calification of the pulp. Dr. Winder, of Baltimore, spoke of a case where there was an outgrowth of dentine and of the manner in which it had been treated.

The subject of Mechanical Dentistry was then taken up. Dr. Winder made a few brief remarks on his experience with celluloid, at the request of Mr. I. S. Hyatt.

The following resolutions were offered by Dr. Redman:

Resolved, We, the members of the Southern Dental Association, have had a highly pleasant and very profitable excursion on the river, and to various points of interests, this afternoon: therefore,

Resolved, That our heartfelt thanks are given to Mayor Brown, of St. Louis, for the use of the city harbor boat on the occasion.

Resolved, That we are under grateful obligations to the St. Louis members of this Association for their grand entertainment on the boat, and for numerous courtesies while in the city, especially in providing the steamboat excursion.

Resolved, That our thanks are due to Mr. A. B. Garrison, vice-president of the Vulcan Iron Works, for showing us through his works, where the process of making rails and of running pig iron was seen; to Colonel R. M. Renick, vice-president of the board of water commissioners, for conducting us through the St. Louis waterworks at Bissell's Point, where some of the largest machinery in the United States was seen; and to Captain John Coale, master of the city harbor boat, for his excellent management of the boat in the trip of twenty-eight miles on the river.

Accepted, with an amendment extending the thanks of the Association to the various newspaper offices for their courtesies.

The Convention then adjourned, to meet again Friday morning at 9 o'clock.

FOURTH DAY'S PROCEEDINGS.—MORNING SESSION.

The session was called to order at 9 o'clock, Dr. Arthur in the chair.

The first subject on the docket was that of the Celluloid base. Mr. Hyatt, secretary of the company which manufactured that substance, made brief remarks in reference to it, that were highly commented upon by several of the members. A paper on the manner of treating exposed nerves, with creosote, and oxychloride of zinc, was next read, and was received very favorably by the Association.

Col. H. S. Knight, passenger agent of the Chicago and Alton R. R., learning that some of the members intended making a trip to Detroit, submitted to them a proposition wherein he agreed to supply round trip tickets at $20, the regular rates being $16.50 each way. The proposition was considered a very liberal one, and was taken under advisement.

"Dental Education" was the next subject introduced for discussion. Dr. Cobb spoke of the great importance of the subject and said that the progress of the profession to the elevation desired could never be attained without more attention being given to it in an educational point of view.

Dr. Arthur considered it a subject well worthy of the most serious attention. He thought that after this time it would be too late to attend to the question, and said that it was a duty we owe to ourselves as well as to the public to see that those who enter the profession are better educated than, as a general thing, they are.

If there are no institutions where a good education of this kind can be obtained, then it becomes the duty of the profession to see that such are established. Dr. Arthur spoke at length on the subject, saying that the dental profession was as capable of furnishing able professors as can be found anywhere in the world. The doctor desired to establish a dental institution, provided he could find men of the profession who would co-operate with him.

Dr. Morgan, of Nashville, was of the opinion that the dental colleges of to-day were far superior to those of twenty years ago. He saw no way of establishing a college of the kind unless it was very heavily endowed, so that the professors could receive pay enough to allow them to devote their entire time to instruction.

Drs. Knapp and Walker, of New Orleans, gave their views of the subject, expressing themselves in very forcible terms against the practice of issuing diplomas as fast as possible, with no regard to the amount of education imparted, and with sole regard to financial considerations.

The balloting for officers of the Association for the ensuing year was then in order. Dr. J. R. Walker, of New Orleans, was elected President; Dr. Isaiah Forbes, first Vice President; Dr. Redman, of Nashville, second Vice President; Dr. Freeman, of Nashville, third Vice President; Dr. Judd, of St. Louis, Corresponding Secretary; Dr. J. F. Thompson, of Fredericksburg, Va., Recording Secretary, and Dr. J. Hall Moore, of Richmond, Treasurer.

The following executive committee was appointed: Drs. Arrington, Morgan, Knapp, McKellops and Henkel.

Dr. Walker moved that the time of the next meeting be changed from Mardi Gras day to the last Tuesday in April. Lost.

Dr. Park presented the following resolutions:

Resolved, That a vote of thanks be extended to the press of St. Louis for the kind and efficient manner in which they have reported the proceedings of this Association.

Resolved, That a vote of thanks be given to the officers of the Association for the able manner in which they have administered the affairs of their respective offices. Unanimously adopted.

Dr. Arthur, on retiring from office, stated that he was greatly indebted to the Association for the interest shown during the proceedings, and that he always endeavored to perform the duties of his office to the best of his ability.

Dr. Thompson moved that Dr. King should escort the newly elected officers to their seats. Adopted.

Dr. King seated Dr. Walker, who again extended his thanks, and the thanks of the section of the country he represented, for the kindness shown him.

Dr. Forbes, first Vice President, when called upon for a speech, remarked, "Brevity is the soul of wit," and took his seat. Dr. Redman could only repeat the same remark made by Dr. Forbes. Dr. Walker spoke of Dr. Forbes' remark, and hoped it would be a lesson to the members and they would profit by it. Dr. Arrington was glad Dr. Walker had come to that conclusion. Drs. McReynolds and Henkel were appointed delegates to the American Dental Association. Dr. McKillops invited the members of the profession present to attend the session of the Mississippi Dental Association to be held at Cincinnati next month.

The Convention then adjourned to meet at Memphis, Tenn., on Mardi Gras day.

